# Cervical cesarean damage as a growing clinical problem: The association between in-labour cesarean section and recurrent preterm birth in subsequent pregnancies

**DOI:** 10.1371/journal.pmed.1004497

**Published:** 2024-12-12

**Authors:** Laura van der Krogt, Andrew Shennan

**Affiliations:** Department of Women and Children’s Health, School of Life Course and Population Sciences, King’s College London, St Thomas’ Hospital, London, United Kingdom

## Abstract

Laura van der Krogt and Andrew Shennan discuss the potential underlying role of cervical caesarean damage in the association between in-labour caesarean section and recurrent preterm birth in subsequent pregnancies.

## Teaser

Increasing evidence supports an association between in-labour cesarean section and recurrent preterm birth in subsequent pregnancies. This clinically challenging problem may be caused by cervical damage at the time of in-labour cesarean section.

The rate of cesarean section has been increasing globally over several decades. Between 1990 and 2014, there was an absolute increase in cesarean section rate of 12.4%, translating to 1 in 5 women delivering by cesarean section worldwide [1]. In England, over 1 in 3 women deliver by cesarean section, and 24% of all deliveries are by emergency cesarean section, of which 5% are performed on women at full cervical dilatation [2,3]. Overall, the rates of full dilatation cesarean section are on the rise, with an increase of 44% in 10 years reported in North America [4]. It is considered that changes in professional training and practice, increasing fear of litigation, as well as social, and cultural expectations are all contributory factors to the rise in in-labour and full dilatation cesarean section [1].

Cesarean section is a necessary and life-saving procedure when complications arise during pregnancy and labour, but it can have important implications for future pregnancies. Emergency cesarean section, particularly when performed late in labour, is associated with preterm birth in subsequent pregnancies. There is a body of evidence, primarily from observational studies, which has shown an association between delivery by in-labour cesarean section and an increased risk of subsequent mid-trimester loss (defined in the United Kingdom as delivery after 13 weeks and before 24 weeks gestation), and spontaneous preterm birth (sPTB) (delivery before 37 completed weeks gestation) [5,6]. Risks appear to be greater with increasing dilatation and are highest when the cervix is fully dilated [5]. For most women who have had an in-labour cesarean section, the risk of preterm birth in subsequent pregnancies remains low (<5%). However, in the small number of women who have a subsequent preterm birth following an in-labour cesarean section, this is more likely to recur in a future pregnancy.

Of note, a recent study in the UK found that, in women with a previous in-labour cesarean section and subsequent preterm birth, the relative risk (RR) of recurrent sPTB was 2.7 (95% confidence interval (CI) 1.87 to 3.87), compared to women who experienced sPTB without in-labour cesarean section as a risk factor [6]. The association is stronger when also including mid-trimester loss, with an RR of 5.65 (95% CI 2.60 to 12.00) for sPTB and delivery earlier than 24 weeks gestation [6]. Among women with previous in-labour cesarean section and subsequent preterm birth in this cohort, 54% had another preterm delivery, which is considerably higher than other groups at high risk of sPTB for other reasons including history of previous sPTB [6].

The association between sPTB, mid-trimester loss and in-labour cesarean section may be related to cervical cesarean damage. The cervix is key to maintaining a pregnancy, and the cervical internal os, which has smooth muscle cells in the pattern of a “sphincter,” is thought to be central to cervical function [7]. It has been suggested that damage to the cervix, particularly at the level of the internal os, may disrupt the integrity and function of cervical tissue in future pregnancies [7].

**Fig 1:**
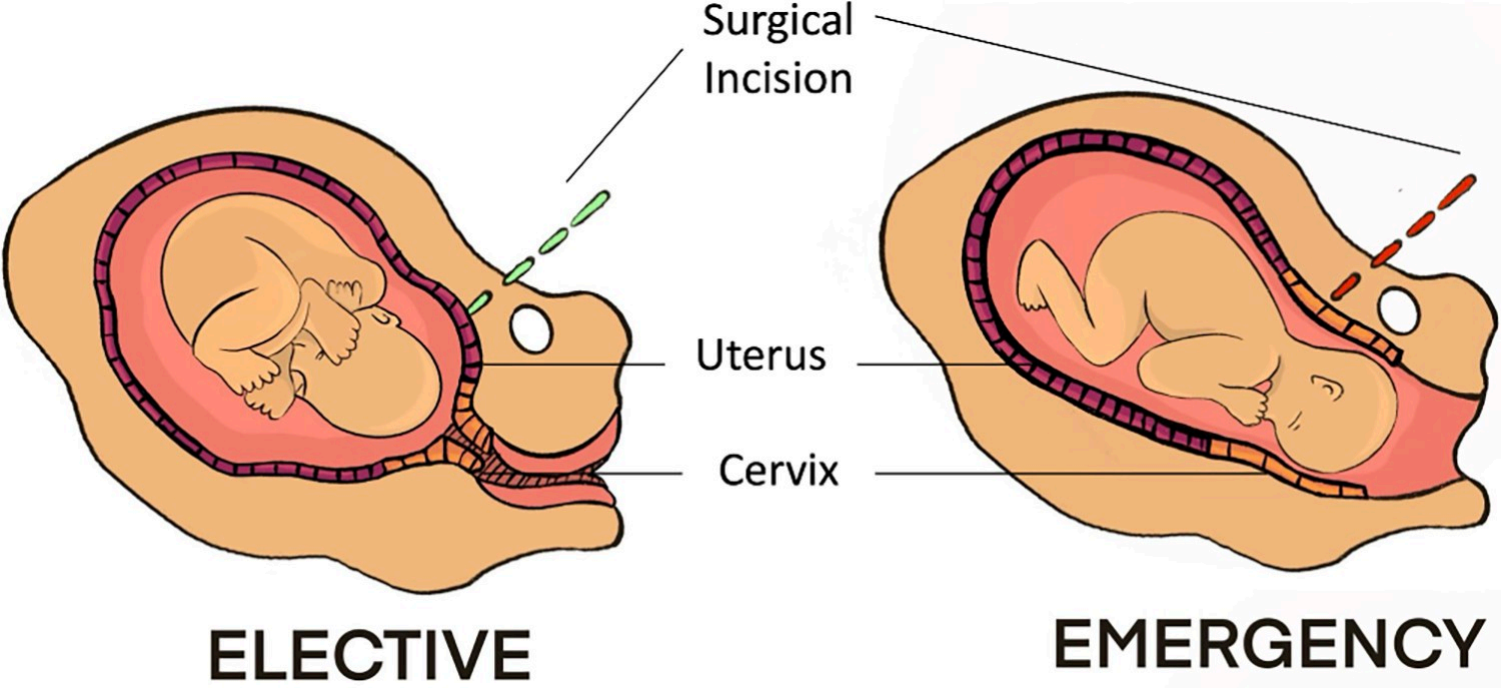
Proposed site of surgical incision at time of elective versus an emergency cesarean section in labour.

At the time of in-labour cesarean section, the surgical incision is more likely to be near or within the cervical tissue due to the physiological changes the cervix undergoes during labour (Fig 1). Moreover, as labour progresses, the fetal head is lower in the maternal pelvis and can be more difficult to deliver, increasing the risk of surgical trauma to the cervix. A recent study demonstrated that the fetal head in a low position in the maternal pelvis at the time of first cesarean section is associated with an increased risk of sPTB below 32 weeks gestation in a subsequent pregnancy (odds ratio (OR) 1.73 (95% CI 1.05 to 2.84)) [8]. Other potential mechanisms include surgical site extensions, suture material at the time of cesarean section, the healing process, and incidence of post-operative infection, all of which may impact upon cervical integrity.

Imaging with transvaginal ultrasound (TVUS) supports the hypothesis of cervical cesarean damage as a possible underlying mechanism. The cesarean section scar can be visualised on TVUS as a hypoechogenic discontinuity of the myometrium in the anterior uterine wall [9,10]. Studies have demonstrated a correlation between cervical dilatation at the time of cesarean section and location of the cesarean section scar, with the scar more likely to be positioned at or below the level of the internal os at advanced dilatation [9]. A recent study illustrated that a cesarean section scar located <5.0 mm above or below the internal os was associated with cervical shortening and sPTB [10].

Cervical cesarean damage may also explain why one of the conventional interventions to prevent sPTB, transvaginal cerclage (TVC), was shown to be less effective in women with a previous in-labour cesarean section, compared to other women at high risk of sPTB [11]. TVC involves placing a suture vaginally between 11^+0^ to 24^+0^ weeks of gestation. It may be history-indicated due to preexisting risk factors such as previous sPTB, mid-trimester loss or cervical surgery, or ultrasound-indicated for a short cervix <25 mm on imaging. In a study of over 200 women receiving TVC, women with a previous in-labour cesarean section were 10 times more likely to deliver before 30 weeks of gestation compared to those with any other risk factor (17/56 versus 5/154) [11]. In this study, 46% of women receiving a TVC with a previous in-labour cesarean section had either a mid-trimester loss or sPTB [11].

Transabdominal cerclage (TAC) may be a more effective treatment option in women with a previous in-labour cesarean section. TAC is placed at the level of the internal os and is currently reserved for women who have had a failed TVC. TACs are placed by laparotomy or laparoscopy, often prior to pregnancy. In women with previous in-labour cesarean section, the suture is positioned above the area of cervical cesarean damage, which can be identified at the time of surgery. A retrospective cohort study demonstrated that TAC was associated with considerably fewer cases of sPTB at <30 weeks gestation compared to TVC (OR 0.09 95% CI, 0.00 to 0.59; *p* = 0.008) [12].

The association between mid-trimester loss and sPTB following an in-labour cesarean section indicates a growing clinical problem. The increasing number of in-labour cesarean section needs to be addressed through supporting professional training in the management of labour and instrumental delivery. Further research is required to determine the exact underlying mechanisms of cervical damage during in-labour cesarean sections and methods to minimise injury to the cervix intra-operatively. There is a need to identify optimum management strategies for this cohort of women who may be at higher risk of mid-trimester loss and sPTB, particularly as standard treatments appear to have a high failure rate. Optimal imaging protocols need to be established and the value of interventions once risk has been established needs to be evaluated. Cesarean sections are the most common surgery in the world, affecting nearly one quarter of women. Cervical cesarean damage and its potential implications for future pregnancies need to be recognised and considered in management plans and shared decision-making between women and clinicians.
